# A Meta-Model Integration for Supporting Knowledge Discovery in Specific Domains: A Case Study in Healthcare

**DOI:** 10.3390/s20154072

**Published:** 2020-07-22

**Authors:** Andrea Vázquez-Ingelmo, Alicia García-Holgado, Francisco José García-Peñalvo, Roberto Therón

**Affiliations:** 1 GRIAL Research Group, Computer Science Department, University of Salamanca, 37008 Salamanca, Spain; aliciagh@usal.es (A.G.-H.); fgarcia@usal.es (F.J.G.-P.); 2 VisUSAL, GRIAL Research Group, Computer Science Department, University of Salamanca, 37008 Salamanca, Spain; theron@usal.es

**Keywords:** model-driven development, dashboard, meta-model, knowledge management, healthcare, technological ecosystem, health ecosystem, meta-model integration

## Abstract

Knowledge management is one of the key priorities of many organizations. They face different challenges in the implementation of knowledge management processes, including the transformation of tacit knowledge—experience, skills, insights, intuition, judgment and know-how—into explicit knowledge. Furthermore, the increasing number of information sources and services in some domains, such as healthcare, increase the amount of information available. Therefore, there is a need to transform that information in knowledge. In this context, learning ecosystems emerge as solutions to support knowledge management in a different context. On the other hand, the dashboards enable the generation of knowledge through the exploitation of the data provided from different sources. The model-driven development of these solutions is possible through two meta-models developed in previous works. Even though those meta-models solve several problems, the learning ecosystem meta-model has a lack of decision-making support. In this context, this work provides two main contributions to face this issue. First, the definition of a holistic meta-model to support decision-making processes in ecosystems focused on knowledge management, also called learning ecosystems. The second contribution of this work is an instantiation of the presented holistic meta-model in the healthcare domain.

## 1. Introduction

At present, society is changing. Three main concepts are used to refer to these changes, information society, knowledge society, and digital society. These concepts are strictly related, and sometimes authors use them as synonyms. Each concept emphasizes an aspect of society. In particular, the digital society concept is used to put a focus on how digital technologies have an digitalization impact on today’s society, culture and politics. According to Dufva and Dufva [1], digital technologies are entangled in the structures of society in many different, complex and even contradictory ways. In this context, the concept of information society is used above all when dealing with technological aspects and their effects on economic growth and employment [2]. 

On the other hand, the concept of the knowledge society emphases that knowledge management is available for most people due to the unlimited access to information. The core element is not the technology but the ability to identify, produce, process, transform, disseminate and use the information to build and apply knowledge for human development [3]. Knowledge management requires training, apprenticeships and other more costly forms of transmission, while information can generally be reproduced at minimal cost [4]. 

According to Castells [5], what characterizes the current technological revolution is not the centrality of knowledge and information, but the application of such knowledge and information to knowledge generation and information processing/communication devices. Knowledge has become a crucial element for development, given its power as a strategic factor for building new policies, planning new actions and fostering innovation within organizations. Knowledge management is considered a sustainable competitive advantage [6], so organizations expend part of their resources on building their capacity to transform and transfer new knowledge continuously over time [7].

However, knowledge is not only present physically (i.e., in documents or books), it is also present in employees and the different processes carried out at organizations. Eraut [8] defines this type of knowledge as the cognitive resource which a person brings to a situation that enables them to think and perform. This knowledge may be either explicit or tacit, and it is the tacit form which is more difficult to codify and reproduce. This tacit knowledge includes experience, skills, insights, intuition, judgment and know-how. According to [6], knowledge management processes must be able to support the transfer of tacit knowledge to explicit knowledge and vice versa. This scattered nature of knowledge makes its management a complex and crucial task. Additionally, the increasing number of information sources and services, such as intelligent devices, sensors embedded in the environment and the Internet-of-Things (IoT), increase the amount of information available, and therefore there is a need to transform that information into knowledge. 

Although knowledge management is one of the key priorities of institutions and companies, according to Davenport et al. [9], the implementation of knowledge management processes within an organization could be expensive, meaning that not all organizations have the capability and the resources to profit from their knowledge. Also, the different domains present different challenges. For example, in the healthcare domain, there is a trend in developing sensor-based applications for monitoring well-being or health conditions and to trigger alarms sent to relatives or care centers [10,11]. In particular, the development of ambient assisted living platforms and systems has increased in the last decade due to the alarming numbers of growing elderly population [11].

Over recent years, software ecosystems have emerged as a technological solution to support information and knowledge management in different contexts. Manikas and Hansen [12] define the software ecosystem as the interaction of a set of actors on a common technological platform that results in a series of software solutions and services. Other authors [13,14,15] refer to ecosystems that have a central software system or platform that provides a set of core functionality and offers the tools for users or developers to contribute services and software and hardware components to extend the functionality of the ecosystem. Institutions adopt a software ecosystem strategy to expand their organizational boundaries, share their platforms and resources with third parties, and define new business models [12,16]. 

There are many terms to describe this type of solution, though each one has distinctive characteristics [17]. In particular, the technological ecosystem concept emphasizes the human factor as an inherent part of the ecosystem in order to develop solutions with evolutive capabilities focused on supporting knowledge management in heterogeneous contexts. Technological ecosystems provide a general framework that allows defining and developing any type of technological solution, describing how data and information are shared between the ecosystem actors and how those actors interact with each other [18]. One of the main strengths of technological ecosystems is that when their components collaborate, they exploit all of their benefits, obtaining the most out of their functionalities to provide elaborate services. However, there are some challenges associated with the definition and development of these solutions. In particular, challenges associated with decision-making processes related to knowledge management, as well as the changes that continuously occur in any organization.

Furthermore, different terms are used to name technological ecosystems, depending on the domain they are focused. For example, if knowledge management is directed on supporting learning processes, the term learning ecosystem is used. A learning ecosystem is not only a technology, it is a community, with educational methods, policies, regulations, applications and work teams, which can coexist so that their processes are interrelated, and their application is based on the physical factors of the technological environment [19].

In the health sector, technological ecosystems in care and assistance share common characteristics in terms of providing technological means to a community of users (patients, relatives, caregivers, doctors, etc.) in order to involve all of them in providing better care and assistance-related services [20]. For this domain, information dashboards can be very powerful to exploit knowledge within healthcare technological ecosystems. Information dashboards allow through visual analysis [21,22] the identification of patterns, outliers, relationships, etc., within data, enabling the generation of knowledge.

However, dashboards need to be adapted to their audience [23], to specific data domains, and to the tasks that will be performed to analyze these data, among other factors. This is necessary because users have different mental models [24], goals, experience, literacy, domain knowledge, and so on. [25,26,27,28,29,30,31,32,33,34], making the design process of a dashboard a complex task where the elicitation of requirements can be seen as the backbone process, as it will drive the subsequent phases and decisions regarding the configuration and design of the tool.

A meta-modelling approach can be employed to ease this process. It is possible to obtain a general structure of dashboards that can be instantiated and adapted to any kind of contexts, data domains or audiences by abstracting the common and primitive elements of information dashboards. But not only dashboards’ technical elements need to be taken into account during the meta-modelling process. As will be detailed, the inclusion of dashboard users and their requirements as elements of the meta-model enables the integration of this meta-model as a part of technological ecosystems, specifically, learning ecosystems, providing support to discover knowledge and support decision making processes [35].

This work provides two main contributions. First, the definition of a holistic meta-model to support decision-making processes in ecosystems focused on knowledge management, also called learning ecosystems. This meta-model integrates two meta-models defined in previous works: a learning ecosystem meta-model to support the definition of learning ecosystems based on open source software [36]; and a dashboard meta-model to support the analysis of information in order to transform implicit knowledge into tacit knowledge.

The second contribution of this work is an instantiation of the presented holistic meta-model in the healthcare domain. More specifically, an instantiation of a dashboard to support caregivers in order to be included in the eHealth technological ecosystem has been carried out [37]. This case study aims to present the holistic meta-model as a flexible solution able adapt to different actors and contexts. Knowledge management processes not only directly or indirectly affect patients and their relatives, but also healthcare professionals. In particular, there is a need to improve knowledge management processes related to dependent persons due to the aging of the population, with special emphasis in developed countries. The number of persons over 60 years is growing faster than all younger age groups [38], and these numbers have an impact on the cost of care and the resources needed for this population.

The rest of this paper is organized as follows. Section 2 presents related works. Section 3 outlines the methodology followed to develop the meta-models. Section 4 describes the learning ecosystem meta-model, the dashboard meta-model and their integration. Section 5 outlines the architecture of the technological ecosystem in which the dashboard will be included. Section 6 presents the dashboard instantiation. Finally, Section 7 and Section 8 discuss the results and depict the conclusions derived from this work. 

## 2. Related Work

According to a systematic review [20], proposed or developed health ecosystems tend to have problems when it comes to being deployed in the real world. Nevertheless, countries and different organizations have dedicated strong investment to the development of health ecosystems in the last decade, looking for new innovative solutions that could alleviate the increasing economic requirements of the health sector [39]. According to other studies about the trends in the development of technological ecosystems focused on health, it can be observed that web-based ecosystems are the most frequently developed, but sensors are present in most of the eHealth ecosystems analyzed [39,40]. Even though there are solutions for different domains inside the health sector, there is a lack of flexible solutions able to adapt to different actors and contexts. Furthermore, the trend in developing platforms and systems that integrate different sensors presents an additional challenge. As data is constantly streamed, the classic information pull (gather, analyze, decide) has to be complemented by a real-time business process [41].

In this context, dashboards are one of the most useful tools for generating knowledge about different data domains. Also, dashboards are popular solutions to exploit the data provided by sensors embedded in the environment, intelligent devices and the IoT [42,43].

The automatic generation of information displays, whether dashboards or single-visualizations, has become a research area of interest given the benefits that these generative pipelines could yield. In fact, different methods have been researched to achieve efficient development processes of these powerful tools, from configuration wizards to software engineering paradigms, among other methodologies [44].

In fact, different applications of the model-driven development to the dashboards’ domain can be found in the literature. In [42], a meta-model is employed to visualize sensor data through a composition-based approach. On the other hand, in [45], two meta-models are presented to tackle the automatic generation of visualization and assist non-expert users during the selection process. One of the meta-models describes the user requirements through goals and tasks, and the other meta-model describes the structure of the potential data visualizations. Finally, in [46], a model-driven approach is employed to translate the users’ requirements into a set of suitable visualizations based on a skyline-based technique and design guidelines.

As introduced in the first section, the holistic meta-model presented in this work merges two previously developed meta-models: a learning ecosystem meta-model [36] and a dashboard meta-model [47,48]. This dashboard meta-model has a finer grain than the aforementioned meta-models present in the literature, allowing more sophisticated combinations to obtain a wide variety of dashboard displays.

## 3. Materials and Methods

A model-driven development approach was employed to build the meta-models. Model-driven development (MDD) [49,50] allows separating the data and the operations specification of the system from lower-level details, like the technical aspects related to a specific program language or platforms. This methodology increases the reuse of components (thus decreasing the development time), but also the reuse of knowledge because the structures and relationships identified during the development of the meta-model can evolve to obtain better solutions.

The Object Management Group (OMG) proposal uses model-driven architecture (MDA) as a guideline to implement the MDD approach. This architecture provides a framework for software development in which the process is driven by models that describe and define the target system [51]. The main difference between MDD and MDA is that MDA determines a set of standards to develop the approach, such as meta-object facility (MOF), unified modeling language (UML), Extensible Markup Language (XML) metadata interchange (XMI) and query/view/transformation (QVT).

**Figure 1:**
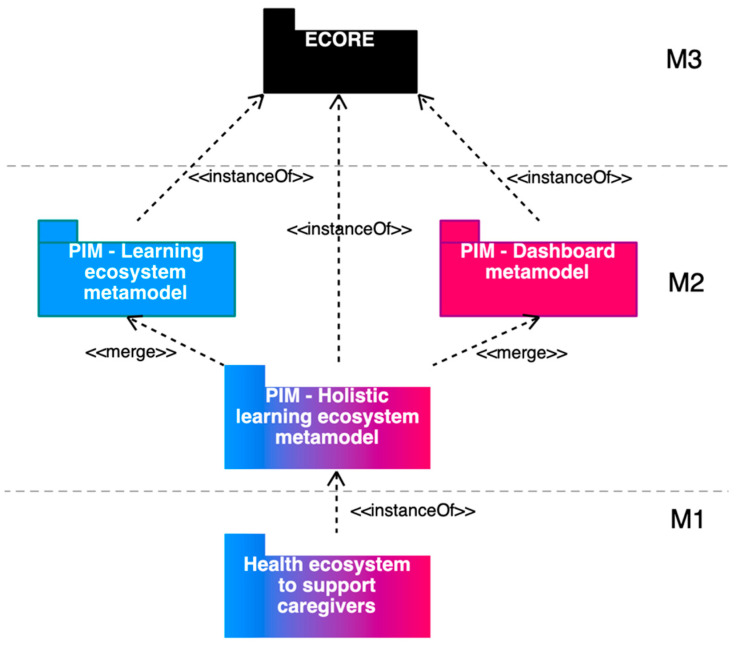
Methodology employed to integrate the learning ecosystem meta-model and the dashboard meta-model organized in the four-layer meta-model architecture of the Object Management Group (OMG).

The dashboard meta-model is also part of the four-layer meta-model architecture proposed by the OMG, in which a model in one layer is used to specify models in the layer below [52]. In particular, the first version of the dashboard meta-model [47,48] was an instance of MOF (i.e., an M2-model), so it can be instantiated to obtain M1-models. This meta-model was transformed in an instance of Ecore [36] using Graphical Modelling for Ecore included in the Eclipse Modeling Framework (EMF), in order to leverage the different features of this modeling framework (Figure 1).

In this case, the dashboard is a part of the learning ecosystem, which is based on a meta-model defined and validated in previous works. The first version of the learning ecosystem meta-model is based on MOF, and the last validated version is an instance of Ecore [36]. This meta-model was developed using a domain engineering approach [53,54], in which similarities and variability points were identified to obtain an abstract picture of the dashboards’ domain in terms of these tools’ elements and features.

The dashboard meta-model is also part of the four-layer meta-model architecture proposed by the OMG, in which a model in one layer is used to specify models in the layer below [52]. In particular, the dashboard meta-model is an instance of MOF (i.e., an M2-model), so it can be instantiated to obtain M1-models. 

The integration of both meta-models is possible because of the fact that both are platform independent models (PIM) in the M2 layer, although one is instantiated from Ecore (learning ecosystem meta-model) and other from MOF (dashboard meta-model). To get the holistic meta-model, the dashboard meta-model was transformed in an instance of Ecore using Graphical Modelling for Ecore included in EMF. Likewise, the final meta-model can be instantiated to obtain a model in the M1 layer.

## 4. Holistic Meta-Model: An Integration of Meta-Models

This section presents the two meta-models that were integrated, the learning ecosystem meta-model and the dashboard meta-model.

### 4.1. Learning Ecosystem Meta-Model

The ecosystem meta-model provides a high-level view of the characteristics that a learning ecosystem has to fulfil to ensure the flexibility of the ecosystem and cover different needs detected in previous studies with real technological ecosystems. In particular, the meta-model definition is based on six real ecosystems for supporting learning processes and knowledge management in different domains (higher education, research, informal learning, youth participation, etc.). Furthermore, the meta-model was used to define real learning ecosystems for public administration, the health sector and knowledge management in PhD programmes.

The objective of the meta-model is to provide a set of guidelines to define learning ecosystems composed of three main elements: software components; a set of components that represent the human factor; and the information flows between the components. The components are black boxes; the learning ecosystem meta-model does not enable capture of the description of a specific component [55]. The meta-model includes Object Constraint Language (OCL) rules which ensure the correct instantiation of a model of the learning ecosystem. Moreover, the guided process is completed with another meta-model, a platform-specific meta-model that provides a pre-selection of open source tools to implement the learning ecosystem. 

**Figure 2:**
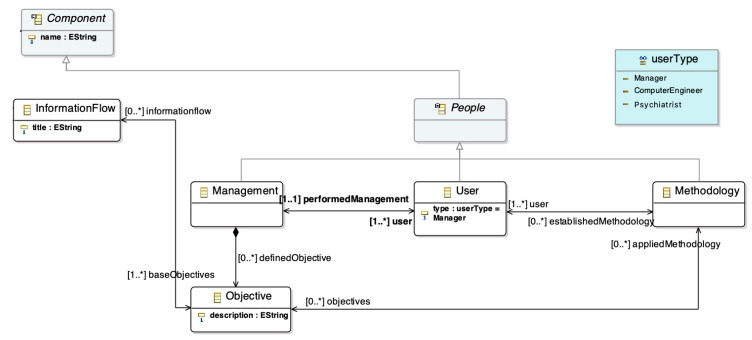
Human factor in the learning ecosystem meta-model. Based on [36].

The learning ecosystem introduces the human factor as something tangible. The human factor is represented through users who have an influence in the other parts of the meta-model. In particular, the human factor is the management processes that define a set of objectives that apply an established methodology (Figure 2). 

**Figure 3:**
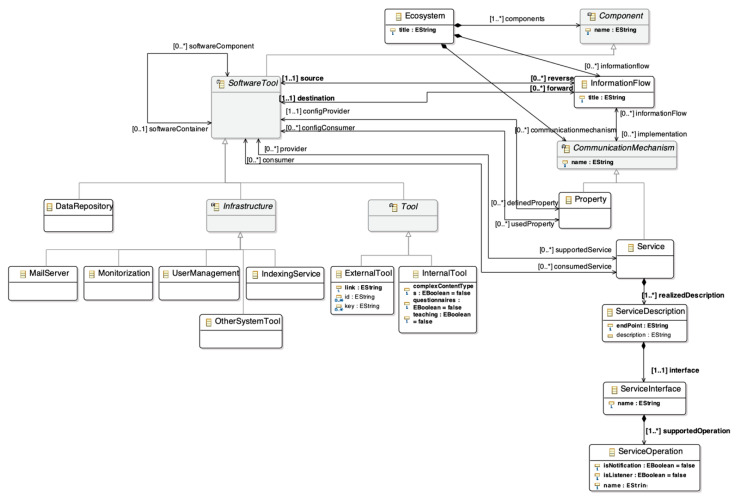
Software components and information flows in the learning ecosystem meta-model. Based on [36]. A high resolution version can be found at https://doi.org/10.5281/zenodo.1066369.

The objectives are used to define the interaction between the different components of the ecosystem. This interaction is implemented through information flows (Figure 3). The ecosystem meta-model takes into account different ways to provide communication between two software tools. For this reason, each information flow has associated communication mechanisms. Moreover, the mechanisms are modelled as a hierarchy to support the evolution of the meta-model, so it is possible to extend it by adding new communication mechanisms.

Finally, the last component of the meta-model is the software tools. They are modelled as a hierarchy organized in the layers of the architectural pattern for learning ecosystems, which was defined as a first step to build the meta-model. This architecture has four layers from bottom to top: infrastructure, static data, services, and presentation. Also, the hierarchy allows the addition of new software types.

### 4.2. Dashboard Meta-Model

The dashboard meta-model represents the high-level and abstracted pieces that compose different types of dashboards and information visualizations. The dashboard meta-model has three main factors that are present in any kind of variant of these tools: the user, the layout, and the components (referring to the content of the dashboard). 

**Figure 4:**
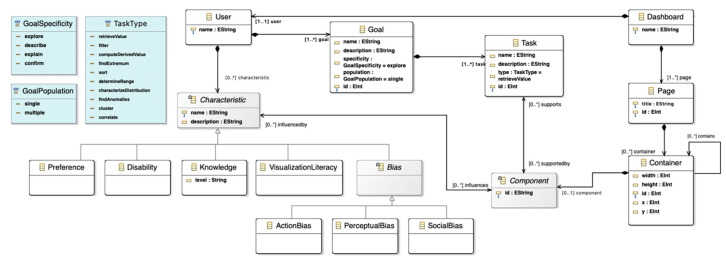
User and layout section of the dashboard meta-model.

**Figure 5:**
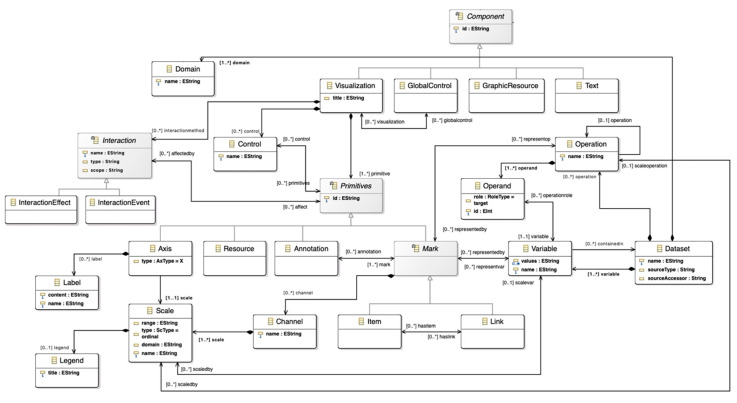
Components’ section of the dashboard meta-model. A high resolution version can be found at https://doi.org/10.5281/zenodo.3625703.

Figure 4 shows an excerpt of the dashboard meta-model containing the three sections mentioned. A detailed view of the components section can be consulted in Figure 5.

While the user section describes who will use the dashboard in terms of the user intent, goals, and profile, the layout and the components define more technical aspects of dashboards.

The meta-model supports the definition of different design questions that are common to any information visualization design process, e.g., how many visualizations will the dashboard hold? How these views will be arranged? What type of visualizations will the dashboard display? What type of interaction patterns will the dashboard support? Will the different views be linked? These are generic questions that will have specific answers depending on the data domain and the target audience of the dashboard. 

Questions like the aforementioned condition crucial design decisions [56], and crucial design decisions need to be driven by the final consumers of dashboards, the users. That is why including the user in this meta-model is essential, because they will be using dashboards to reach insights, to support their decision-making processes or to exploit datasets.

The User class is defined in terms of a series of aspects that can be significant and influential within the design process of a dashboard [57]. Given that, the user entity is decomposed in terms of his or her goals and his or her characteristics.

Firstly, a crucial concept arises; goal. A user must have at least one goal for using a dashboard. Goals, in turn, can be broken down into individual and more specific, low-level tasks. Simple goals can be accomplished by performing a few tasks. However, more elaborated goals might involve several specific and chained tasks, which could involve different data dimensions to support complex decision-making processes [58,59,60].

Users can also have a set of identified characteristics. These characteristics can be diverse, but they need to be well-defined to take them into account while instantiating the meta-models. For example, preferences, disabilities, knowledge about different domains, visualization literacy, and bias are different kinds of characteristics. These characteristics can influence the design process; for example, a user with low visualization literacy might not perceive data correctly through complex visual encodings, so the visualization elements must be adapted properly to account for this user characteristic.

In terms of technical components of the dashboard, several elements are identified. The main components of dashboards are the information visualizations that display data, but also the interaction patterns, controls, graphic resources, or text that complement these visualizations (Figure 3). Information visualizations, in turn, are composed of primitives that encode data variables through channels (i.e., color, size, position, area, etc.). These primitives are the core of information visualizations, because they are the elements that encode the data to be displayed [61,62].

Regarding the data to analyze and visualize, the dataset concept represents the data input in the meta-model. This small piece inside the dashboard meta-model provides a high-level of adaptation. The dataset abstracts the dashboard from the data source. In this sense, the dashboard can integrate different sources such as the interaction generated inside an ecosystem, the information gathered through different sensors or a database provided by the users.

**Figure 6:**
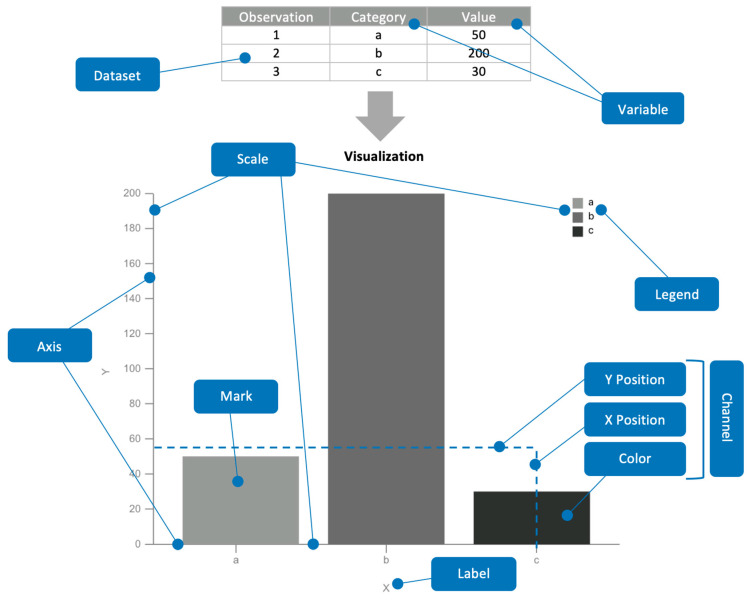
Example of the different elements that compose a visualization (in this case, a bar chart).

Figure 6 shows a practical example of the identification of different parts of information visualizations through the dashboard meta-model classes.

In this example, there are two scales that represent two variables (the “Category” variable through an ordinal scale, and the “Value” variable through a linear scale). The domain of these scales is the set of values from the variables. For instance, the domain of the scale that encodes the X position of the visual marks is the set of values retrieved from the “Category” variable (i.e., ‘a’, ‘b’, and ‘c’). Axes, on the other hand, support the visualization of the scales’ domains.

The visual marks of this visualization are bars with a specific position along both X and Y axes and a specific color based on a color scale that encodes the “Category” variable. Scales map the data values to another specific range of values in order to encode the information, that is why these entities are related both to the dataset variables’ values (to obtain the domain) and to the visual channels or encodings (to encode these values using another specific range of values, like color codes or screen positions).

### 4.3. Meta-Model Integration

The previously presented meta-models have been combined to create a holistic meta-model. The idea behind this holistic meta-model is to leverage the connection and collaboration of the elements from both meta-models. The learning ecosystem meta-model has a lack of decision-making support. While monitorization tools, dashboard and other decision-making tools could be instantiated from *Tools* or *Infrastructure*, the main characteristics of the learning ecosystems would not be taken into account. The present proposal aims to solve this problem.

The dashboard meta-model provides the framework to define an infinite number of solutions based on data analysis and visualization. The main aim of a dashboard is to support the decision-making process, so the integration of the dashboard meta-model with the learning ecosystem will support the definition of learning ecosystems for knowledge management from a holistic point of view.

The integration is possible because booth meta-models are M2-level in the four-layer meta-model architecture of the OMG (Figure 1). Despite this, the granularity of each meta-model is different. In particular, the learning ecosystem meta-model has a high-level of abstraction, in which each software component is represented as a black box. The dashboard would be a black box in the ecosystem meta-model before the integration. On the other hand, the dashboard meta-model defines all the elements inside the dashboard component, the level of abstraction minor compared to the ecosystem meta-model.

The integration of the two M2-level metamodels is based on the connection between some elements present in both meta-models. It is important to highlight that the definition of both solutions was conducted independently in different periods of time, so the integration process implied a deep analysis of both solutions to find the connection elements that represent the same concept.

Despite the abstraction differences, the human factor plays a crucial role in both meta-models. The learning ecosystem meta-model introduces users and other elements, such as *Management* and *Methodology*, to represent the human factor at the same abstraction level as other elements in the ecosystem. Regarding the dashboard meta-model, it is necessary to include users in the proposal because they are the drivers and consumers of the displayed data. Moreover, the characteristics of the users, such as *Preference*, *Disability*, *Knowledge*, or *Bias* are used as an input to instantiate the dashboard.

On the other hand, there are two crucial elements shared in both meta-models too. The dashboard *Goals* (within the dashboard meta-model) are represented as *Objectives* within the learning ecosystem meta-model. These elements are represented by a set of *Tasks* and *Information Flows*, respectively. The relevance of these entities is that they are the core of the meta-model, because they frame the required components to achieve the goals or objectives set.

**Figure 7:**
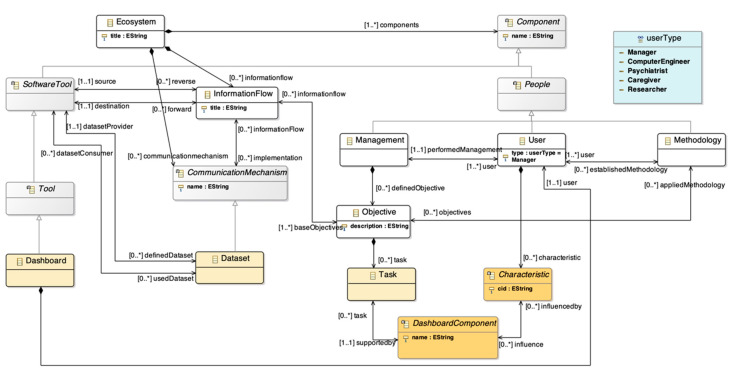
Holistic meta-model: the integration of the learning ecosystem meta-model with the dashboard meta-model.

Figure 7 shows the connection between both meta-models. The dashboard *Goal* is merged with *Objective*. The connection between *Goal* and *User* in the dashboard meta-model is replaced by the association between *User* and *Objective* through *Management*. In this sense, the management decisions in the ecosystem define all the goals that support the definition of the dashboard. 

Regarding the integration of *Dashboard*, the main class of the dashboard meta-model which contains all the elements in the meta-model, it is connected with the learning ecosystem meta-model through a subclass of *Tool*. Besides, the connection between *User* and *Dashboard*, which has a strong impact on the dashboard meta-model, is included in the proposal. The information flows and tasks are different concepts, so it is not possible to merge them. For this reason, the *Task* entity is included in Figure 7. 

On the other hand, a new communication mechanism is included to implement the information flows, the *Dataset*, as a way to represent the integration between the dashboard and other software tools in the learning ecosystem. Also, the dashboard *Component* has been renamed as *Dashboard Component* to distinguish it from the learning ecosystem *Component*. 

Finally, the connection between dashboard *Characteristic* and *User* appears in the new proposal. Tasks are supported by the dashboard’s elements, that are also influenced by the user characteristics to match his or her information requirements.

## 5. Healthcare Ecosystem for Caregivers

The aim of the technological ecosystem for caregivers is to support the learning and knowledge management processes to develop and enhance the caregiving competences both at home and in the care environments of formal and informal caregivers [37]. In particular, the ecosystem allows psychoeducation [63] to be provided to dependent persons and informal caregivers in order to alleviate the physical and mental health problems that they suffer, such as work overload, depression or anxiety.

**Figure 8:**
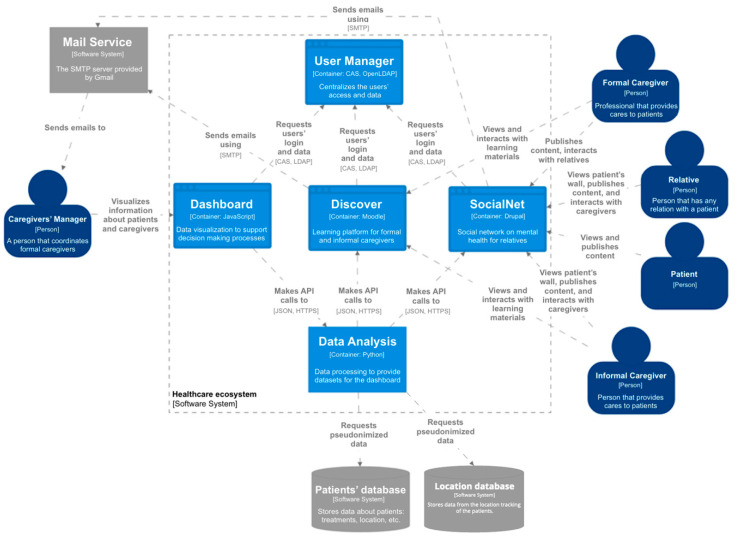
Healthcare ecosystem architecture in C4 Model.

The ecosystem makes it possible to provide remote access to different services. It is composed of a set of software components (Figure 8) and based on a set of management and methodological input streams—a business plan, a training plan, and a medical protocol. First, it provides remote teaching-learning environments to support both informal and formal caregivers. Through *Discover*, psychoeducation is accessible to these profiles, so they can obtain psychological support and answers to the questions that arise daily during their care duties, as well as information, advice, and guidance.

Second, *SocialNet* is an online tool that provides a private social network composed of a set of private and safe areas, called walls, for each patient [64]. The main users are the relatives of the patients and their caregivers. In some cases, patients can also access to *SocialNet* to publish their activities and view the contents published by their caregivers and relatives. Finally, the caregivers’ managers can access to the social network, but only to manage which caregivers control a patient’s wall (this relationship is not represented in Figure 8 to avoid lines crossing the whole system).

Third, the *Dashboard* is a tool to support decision-making processes. In particular, it is focused on supporting caregivers’ managers to make decisions about the workload of the caregivers, and the activity of the patients based on insights from the different components of the ecosystem.

Moreover, two software components provide support to other components, the *User Manager* that centralizes the users’ data management and the access, and a tool to support the analysis of the data get from the *Discover*, *SocialNet*, and external database with medical and personal information about the patients. Moreover, the ecosystem is constantly evolving; the latest update has been the integration of patients’ location data through a NoSQL (non-relational) database that stores them. The *Data Analysis Support* provides the datasets for the dashboard component.

## 6. Meta-Model Instantiation: A Dashboard for Supporting Caregivers

To illustrate the integration of the two meta-models, we present an instantiation of a dashboard for supporting caregivers based on the healthcare ecosystem described in the previous section. This dashboard gives support to achieve different information goals related to the management of the caregivers. Specifically, two main goals were identified:

- To analyze the relationship between the attention given by relatives and the patient’s health;
- To gain insights about the workload of the caregivers.

Caregivers’ managers will use this information to make decisions regarding the caregivers’ organization to balance their workloads and to understand if some patients need more attention. Three visualizations were proposed to support these goals. 

The component selected to support the first goal is a scatter chart that displays information regarding the patient’s health and the attention given by their relatives. On the other hand, the caregivers’ workload can be visualized in different ways and can involve different variables. In this case, two components have been selected to support the second goal: a heat map and a treemap to let managers identify patterns or relevant data points regarding the caregivers’ distribution along time and among patients.

**Figure 9:**
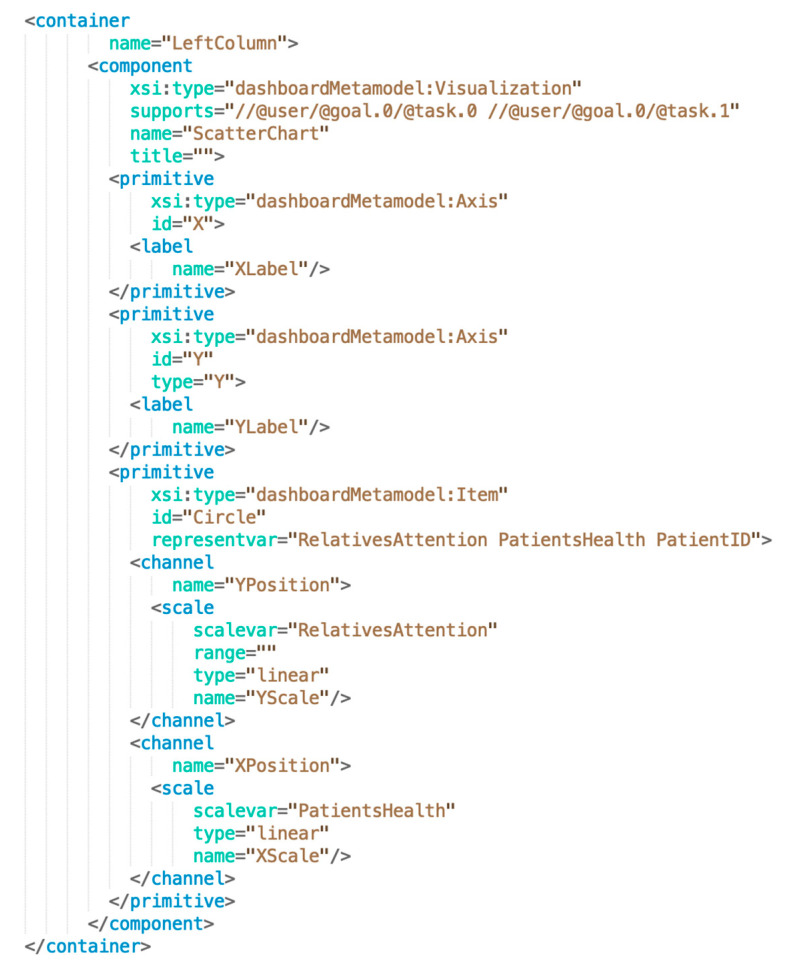
Fragment of the Extensible Markup Language (XML) metadata interchange (XMI) instance file containing the information regarding the scatter chart component of the dashboard.

The instantiation of the dashboard was performed through EMF, obtaining a M1-model with specific values for the target dashboard. Figure 9 shows an excerpt of the XMI file containing the instance structure and values of the scatter chart visualization component.

This instance is then handed to a custom Python dashboard generator which “translates” the XMI structure into a set of software components. These components are tailored from a set of core assets developed following the SPL paradigm [65]. 

The dashboard generator takes the XMI files defining the dashboard as an input, and, depending on the definition of the different visualization, it will select the specific core assets, combining them to obtain the final visualization. For example, to build a bar chart, the generator would compose and configure two axes (X and Y) and visual marks with rectangular shape that encode some variable through their length. The different code fragments that hold the functionality of each primitive element are combined with the support of a template engine [66].

**Figure 10:**
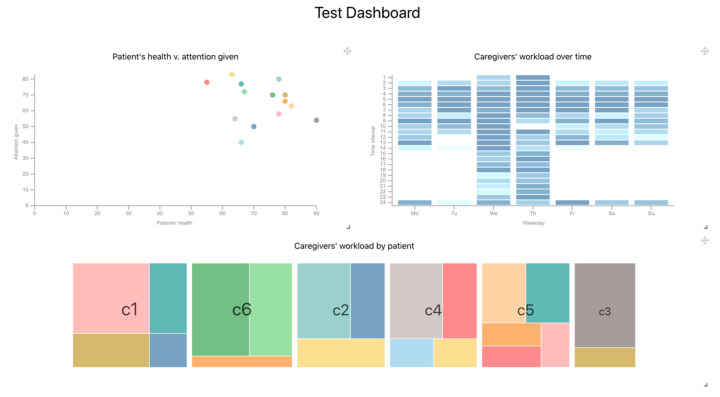
Screenshot of the dashboard generated through the XMI instance. Three visualizations are shown: a scatter plot that display potential relationships between the patients’ health and the attention given to them; a heatmap that display the aggregated workload of the caregivers during the different days and hours of the week; and a treemap that display the individual workload of each caregiver.

The result of the generation process is the front-end’s React source code of a fully functional dashboard with three components that support the initially defined user goals: a scatter plot that shows potential relationships between the patients’ given attention and their health; a heatmap that shows the caregivers’ workload during different time intervals; and a treemap that shows the proportions of the caregivers’ workload by patient (Figure 10). The dashboard receives the data through the configuration of the data sources that are available within the healthcare ecosystem.

## 7. Discussion

Knowledge management is a complex task and requires good planning to obtain benefits. Gathering the best available knowledge is not always easy; organizations must understand who holds crucial knowledge, otherwise knowledge management loses all importance [67]. While information can generally be reproduced for minimal costs, knowledge reproduction requires training, apprenticeships, and other more costly forms of transmission [4]. The transformation of tacit knowledge into explicit knowledge implies not only an investment in technology but also the analysis and definition of the knowledge management processes that allow that transformation. According to [67], creating knowledge is a process of organizing data into information that can be analyzed and used to make educated decisions. In this context, the technological ecosystems, specifically the learning ecosystem that is directly focused on knowledge management, provide a framework to develop flexible solutions in which technology is used to establish information flows that support the transformation of knowledge into tangible information inside the organization. However, the main innovation of this type of technological solution is not the technology itself, but the introduction of the human factor at the same level as the software components. Despite this, the learning meta-model has a lack related to the decision-making processes, which are vital to support the transformation of tacit knowledge into explicit knowledge.

Including information dashboards as software tools within the learning ecosystem meta-model enables not only knowledge management but also knowledge discovery. Information dashboards provide different methods of presenting the generated data in an understandable manner, supporting users to identify patterns, relationships, and interesting data points, as well as to reach insights regarding these data.

The development of the presented holistic meta-model is supported by some conceptual classes present in both meta-models. One of these classes is the *User*. The user represents the human factor in both domains. This entity is crucial because it will define (through goals and objectives) the ecosystem’s and dashboard’s components. This is why the ecosystem’s *User* and the dashboard’s *User* are merged, obtaining a *User* entity that is related to different objectives (goals) that can be decomposed by different tasks that a series of dashboard components will support. The *User* also has characteristics that will influence these components (as presented in the dashboard meta-model).

On the other hand, dashboards are modeled as specializations of *SoftwareTool*, because the dashboard, in addition to supporting knowledge discovery, is also a tool that is part of the ecosystem. 

To illustrate this approach, a dashboard has been generated within the healthcare context. The dashboard for supporting caregivers is part of the healthcare ecosystem. It aims to provide tools based on visual analysis to support decision-making processes related to the workload of caregivers and the patients’ situation. The dashboard should be adapted to the different needs of its users. Furthermore, the medical contexts in which the ecosystem could be implemented are very different, so the ecosystem should be adapted to these different contexts.

The dashboard meta-model provides a “template” for generating concrete dashboard solutions and supports the instantiation of fine-grained features regarding visualizations by distinguishing basic primitives that can be combined to build any type of chart. This meta-model has been instantiated to obtain a concrete model for the presented healthcare context. Three visualizations have been selected and instantiated to support the caregivers’ managers’ goals and decisions with data. However, the dashboard meta-model provides freedom to modify the whole dashboard structure in order to adapt it to other data sources, contexts and audiences. In fact, given that the datasets (and thus, the data sources) are represented within the dashboard meta-model, it is possible to combine different data sources (even if heterogeneous) to unify scattered data into a single information display.

The integration of the meta-models presented not only gives relevance to knowledge discovery by including a dashboard as an important part of learning ecosystems, it also frames dashboards in a bigger picture in which they collaborate with different information flows to enable their users to gain insights.

## 8. Conclusions

This work proposes an integration of two meta-models to deal with issues related to knowledge generation and knowledge discovery in learning ecosystems. Specifically, a dashboard meta-model has been merged into a learning ecosystem meta-model to support decision-making processes related to the information flows and management objectives that are present within these ecosystems.

The dashboard meta-model provides a skeleton that can be adapted to instantiate concrete dashboard solutions that enable the analysis and visualization of information (datasets) from different sources, such as databases, users’ interaction, IoT, sensors, etc. To illustrate the meta-model instantiation process, specific dashboard has been generated through instantiation within a healthcare ecosystem for caregivers, which is, in fact, an instantiation of the learning ecosystem meta-model.

On the other hand, the learning ecosystem meta-model solves several problems related to the definition and implementation of learning ecosystems. Learning ecosystems combine tools to support knowledge management. Including an information dashboard within the learning ecosystem address the improvement of knowledge discovery within the ecosystem by providing a tool to visually analyze information flows.

However, although the learning ecosystem was validated and its quality was checked through the framework defined by López-Fernández, et al. [68], it is necessary to validate and apply the same framework to the holistic meta-model proposed due to the fact that the dashboard meta-model has not been fully validated in previous works.

Furthermore, future research lines will involve the refinement of the meta-model through the addition of constraints, rules, and design guidelines to support a generative pipeline, easing the instantiation process to obtain specific products. Finally, it is also necessary to test the instantiated products with users to validate them and verify their usefulness in real contexts.
